# Towards standardized and reproducible research in skin microbiomes

**DOI:** 10.1111/1462-2920.15945

**Published:** 2022-03-07

**Authors:** Matti O. Ruuskanen, Deepti Vats, Renuka Potbhare, Ameeta RaviKumar, Eveliina Munukka, Richa Ashma, Leo Lahti

**Affiliations:** ^1^ Department of Computing, Faculty of Technology University of Turku Turku Finland; ^2^ Department of Zoology, Centre of Advanced Study Savitribai Phule Pune University Pune India; ^3^ Institute of Bioinformatics and Biotechnology Savitribai Phule Pune University Pune India; ^4^ Microbiome Biobank, Institute of Biomedicine University of Turku Turku Finland

## Abstract

Skin is a complex organ serving a critical role as a barrier and mediator of interactions between the human body and its environment. Recent studies have uncovered how resident microbial communities play a significant role in maintaining the normal healthy function of the skin and the immune system. In turn, numerous host‐associated and environmental factors influence these communities' composition and diversity across the cutaneous surface. In addition, specific compositional changes in skin microbiota have also been connected to the development of several chronic diseases. The current era of microbiome research is characterized by its reliance on large data sets of nucleotide sequences produced with high‐throughput sequencing of sample‐extracted DNA. These approaches have yielded new insights into many previously uncharacterized microbial communities. Application of standardized practices in the study of skin microbial communities could help us understand their complex structures, functional capacities, and health associations and increase the reproducibility of the research. Here, we overview the current research in human skin microbiomes and outline challenges specific to their study. Furthermore, we provide perspectives on recent advances in methods, analytical tools and applications of skin microbiomes in medicine and forensics.

## Introduction

Human skin is a complex organ that plays a diverse role in human health and protects us from various environmental exposures (Uberoi *et al*., 2021). Skin maintains balance and homeostasis by forming an active barrier between the internal organs and the external environment (Sotiropoulou and Blanpain, 2012). It provides a habitat for a diverse ecosystem mainly consisting of beneficial (Christensen and Brüggemann, 2014; Delanghe *et al*., 2021; Fang *et al*., 2021) and potentially pathogenic microbes (Findley and Grice, 2014; Hwang *et al*., 2021). As a growth environment, human skin is primarily dry, acidic, nutrient‐poor and cool (Grice and Segre, 2011). In healthy humans, skin temperature averages between 33°C and 34°C indoors, reaching down to 19°C or up to 35°C when exposed to outdoor temperatures between 0°C and 35°C (Lai *et al*., 2017). Even temperatures close to 37°C can be reached in the groin and armpits (Boxberger *et al*., 2021). Thus, the skin is highly selective on the types of bacteria, archaea and eukaryotes that survive and thrive on it (Grice and Segre, 2011; Byrd *et al*., 2018). Stable communities of commensal microbes are primarily established on skin surfaces during the first weeks of a newborn's life, with specific types of communities developing at different body sites (Luna, 2020). However, the communities continue to mature during their whole life, and persisting microbes can be introduced to the community through repeated invasion and colonization until adulthood (Grice and Segre, 2011; Swaney and Kalan, 2021). Through the combination of factors specific to the human skin as an environment, microbial communities on it are unique and distinct from the skin microbiomes of other mammals (Ross *et al*., 2018). Overall, the human skin microbiome represents a complex and dynamic system with significant contributions to the health and development of various diseases and disorders (Byrd *et al*., 2018; Swaney and Kalan, 2021).

## Microbial community assembly on the skin

Like other biological communities, cutaneous microbiota is governed by the constant ecological processes of dispersal, selection, drift and diversification (Kennedy and Chang, 2020). Major phyla of bacteria found on human skin are *Firmicutes*, *Actinobacteria*, *Proteobacteria* and *Bacteroidetes*, although their relative abundances vary between persons, throughout their lifespan (Luna, 2020), and between body sites on an individual (Byrd *et al*., 2018). Correspondingly, the relative abundances of archaea are usually scarce and differ from childhood to old age, but most belong to either phyla *Thaumarchaeota* or *Euryarchaeota* (Moissl‐Eichinger *et al*., 2017; Umbach *et al*., 2021). In addition, fungi, such as genera *Aspergillus*, *Penicillium*, *Candida* and *Cryptococcus*, are commonly found on healthy skin (Leung *et al*., 2016). Species from the genus *Malassezia* are the most commonly occurring fungi on most skin sites and are usually considered part of the healthy skin microbiota (de Hoog *et al*., 2017). In addition, viruses are an integral part of the skin microbiome (Hannigan *et al*., 2015; Liang and Bushman, 2021). Many eukaryotic viruses from families *Polyomaviridae*, *Papillomaviridae*, *Circoviridae*, *Caudovirales Adenoviridae*, *Anelloviridae* and *Herpesviridae* are commonly detected (Foulongne *et al*., 2012; Hannigan *et al*., 2015). The interactions of the virome with other members of the microbiota have been only rarely studied (Hannigan *et al*., 2015; Liang and Bushman, 2021). However, the skin virome can also contribute to the health and disease of the host through the suppressive actions of bacteriophages (Wang *et al*., 2020).

Most of these overarching patterns have been observed when sampling adult subjects. However, the human skin microbiome also changes considerably over time. While microbes have been reliably detected in the human womb (Fricke and Ravel, 2021), the existence of a stable microbiome in the foetal environment before delivery seems unlikely (for a recent discussion on this subject, see Blaser *et al*., 2021). The initial composition of the skin microbiome is thus primarily affected by maternal factors, such as birth mode, which lead to the dispersal of different organisms on the skin (Bokulich *et al*., 2016; Stennett *et al*., 2020). This initial assemblage of microbes is based on different microenvironments of the skin, which vary physicochemically and topographically due to differences in pH, dryness, thickness, folding, the density of hair follicles and glands, and exposure to the environment (Grice and Segre, 2011; Cho and Eom, 2021; Sun and Rieder, 2021). The different layers of skin, from the epidermis to the dermis, and even the adipose tissue, are colonized with unique subsets of microbes in the separate cutaneous compartments (Zeeuwen *et al*., 2012; Nakatsuji *et al*., 2013). This deep penetration of the skin by certain bacteria can lead to immediate immune responses in the host (Nakatsuji *et al*., 2016). In addition, the initial skin microbiome influences the developing immune system and may even affect the person's susceptibility to diseases later in life (Neu and Rushing, 2011; Bokulich *et al*., 2016; Dunn *et al*., 2017). After birth, bacterial diversity of the skin has been observed to increase with age, at least until the pre‐puberty stage (Lehtimäki *et al*., 2017; Zhu *et al*., 2019). During the maturation process, the initially dominant bacteria *Lactobacillales* (especially genus *Streptococcus*) are replaced by the members of both *Actinobacteria* (genera *Propionibacterium* and *Corynebacterium*) and *Proteobacteria* (Lehtimäki *et al*., 2017). Furthermore, the fungal microbiome of the skin appears to be highly unstable and diverse at birth, but its stability increases, and diversity decreases within the first 6 months (Zhu *et al*., 2020). Fungal diversity decreases until puberty and converges into a more stable community dominated by *Malassezia* (Jo *et al*., 2016). The species composition then shifts to obligatorily lipophilic fungi by adulthood (Jo *et al*., 2016; Leung *et al*., 2016; Zhu *et al*., 2020).

**Fig. 1:**
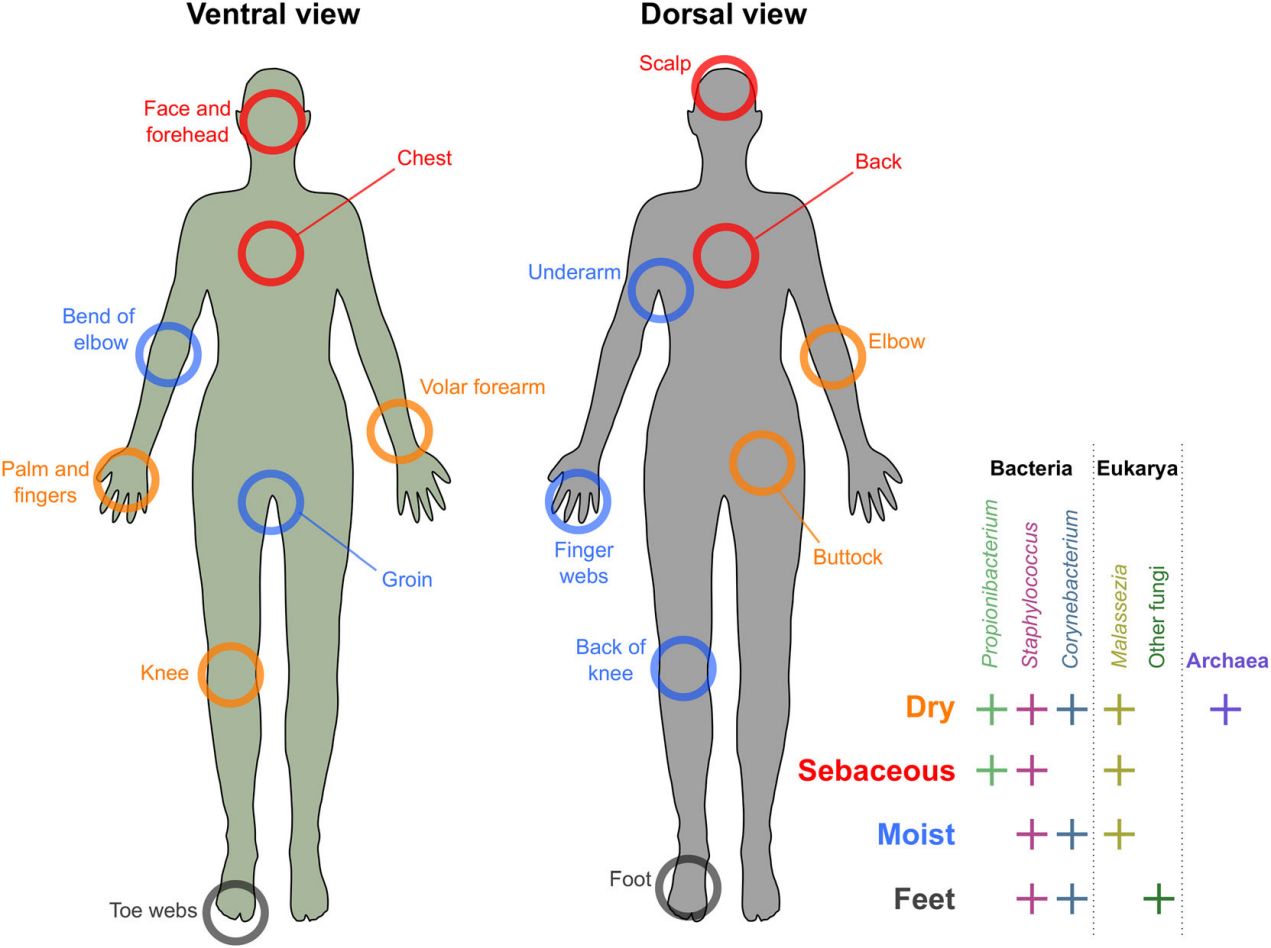
Categorization of common sampling sites for skin microbiomes. The most abundant groups of microbes in each category of sites are shown.

The overall maturation and development of the microbial communities during childhood affects all skin sites or compartments, but the community compositions at individual skin sites also differ considerably. Many microbial species can modify the skin environment through their metabolism, such as *Cutibacterium acnes*, which can break down the sebum lipids into fatty acids and acidify the skin (Flowers and Grice, 2020). The features of the skin site considerably affect its bacterial composition (Byrd *et al*., 2018). Skin sites can roughly be categorized into dry, sebaceous and moist sites (Fig. 1; Byrd *et al*., 2018). The volar forearm is a commonly sampled dry skin site which was used in a number of pioneering studies (Gao *et al*., 2007; Grice *et al*., 2009; Kong *et al*., 2012; Findley *et al*., 2013), likely due to its easy accessibility. These studies have provided convenient reference points for current skin research (e.g. Bjerre *et al*., 2019). For sebaceous sites, the forehead has been similarly easy to sample, and it is an affected area in several inflammatory skin diseases (Dekio *et al*., 2005; Alexis and Talbott, 2021). For moist sites, the bend of the elbow is one of the most often sampled sites, likely also because of its easy accessibility and involvement with, e.g. atopic dermatitis in children (Kong *et al*., 2012; Byrd *et al*., 2018; Alexis and Talbott, 2021).

Generally, dry sites have a high prevalence of genera *Propionibacterium*, *Staphylococcus* and *Corynebacterium*, sebaceous sites are dominated by *Propionibacterium* and *Staphylococcus*, and moist sites have high abundances of *Corynebacterium* and *Staphylococcus* (Byrd *et al*., 2018). While relatively little is known about similar site‐specificity of archaea, their abundance appears to be higher on dryer skin with lower sebum levels and lipid content (Moissl‐Eichinger *et al*., 2017). In the fungal microbiome of the skin, *Malassezia* species dominate throughout the body, except for the feet, where fungal diversity is very high (Grice and Dawson, 2017). However, individual *Malassezia* species can display considerable site‐specificity. *M*. *restricta* is predominant on facial surfaces, and *M*. *globosa* is prevalent in the back (Grice and Dawson, 2017). Biological interactions among microbes and between the host and microbes are crucial for the development and composition of the community. For example, *Staphylococcus aureus* colonization on the skin is prevented by antibacterial compounds produced by *S*. *lugdunensis* (Zipperer *et al*., 2016) and through antimicrobial peptide production of host keratinocytes induced by *S*. *epidermidis* (Iwase *et al*., 2010). These complex inter‐species interactions likely play a role in their uneven distribution in the different microenvironments on the skin (Hernandez‐Valdes *et al*., 2020).

### Inter‐individual differences in skin microbiome composition

In addition to these generic patterns, there is considerable inter‐individual variability in skin microbiome composition. This variability originates from intrinsic factors, including phenotypic differences between hosts, and external factors, such as environmental exposures (Table 1). Many intrinsic factors, such as genetics, sex, ethnicity and age, have been connected with changes in the skin microbiome composition (Si *et al*., 2015; Ying *et al*., 2015; Li *et al*., 2019; Carrieri *et al*., 2021). For example, specific bacterial taxa, such as *Roseomonas* and *Corynebacterium*, likely have high heritability (Si *et al*., 2015). Sex and ethnicity also correlate with differences in bacterial community structure on comparable skin sites (Li *et al*., 2019). Furthermore, the skin microbiome compositions in a small ethnically homogeneous cohort of healthy women could be used to accurately model and predict skin hydration, age, smoking and menopausal status (Carrieri *et al*., 2021). However, the role of interconnected variation in, e.g. skin pH, sebum or sweat production, hormone levels and cosmetics application frequency cannot be ruled out as causes for some of the observed intrinsic variation (Fierer *et al*., 2008; Ying *et al*., 2015).

**Table 1:** Reported intrinsic and extrinsic factors influencing microbiome composition at various body sites.

Factor	Intrinsic (I) or extrinsic (E)	Reportedly affected/sampled skin sites	Associated taxa	References
Genetics^a^	I	Inner wrists	*Brevibacterium*, *Corynebacterium*, *Peptoniphilus*, *Roseomonas*	Si *et al*. (2015)
Ethnicity^a^	I	Multiple body sites	*Corynebacterium*, *Proteobacteria*, *Staphylococcus*	Li *et al*. (2019); Wang *et al*. (2021)
Sex (F)	I	Multiple body sites	*Corynebacterium ↓*, *Propionibacterium ↓*, bacterial diversity ↑	Fierer *et al*. (2008); Ying *et al*. (2015); Li *et al*. (2019)
Age	I	Multiple body sites	*Corynebacterium* ↑, bacterial diversity ↑, archaeal diversity ↑, fungal diversity ↓	Ying *et al*. (2015); Jo *et al*. (2016); Lehtimäki *et al*. (2017); Moissl‐Eichinger *et al*. (2017); Li *et al*. (2019); Zhu *et al*. (2019, 2020)
Contact with people	E	Palms	*Propionibacterium*, oral taxa	Song *et al*. (2013)
Contact with dogs	E	Palms	*Methylophilaceae*, *Actinobacteria*	Song *et al*. (2013)
Contact with environmental microbes	E	Exposed sites	Depending on the environment type, e.g. soil, plants, water	Ying *et al*. (2015); Lehtimäki *et al*. (2017); Nielsen and Jiang (2019); Vandegrift *et al*. (2019)
Environmental conditions (UVA and UVB)	E	Exposed sites	*Cyanobacteria* ↑, *Lactobacillaceae ↓*, *Pseudomonaceae ↓*	Burns *et al*. (2019)
Geography	E	Multiple body sites	Depending on location	Sun *et al*. (2019); Callewaert *et al*. (2020); McCall *et al*. (2020)
Medications (antibiotics)	E	Cheek	*Cutibacterium acnes ↓*, *Cutibacterium granulosum ↑*, bacterial diversity *↑*	Park *et al*. (2020)
Cosmetics	E	Armpits, feet	Depending on body site, sex and compound	Bouslimani *et al*. (2019)
Diet (BMI)	E	Multiple body sites	*Corynebacterium* ↑, bacterial diversity ↓	Brandwein *et al*. (2019)

^a^ The effects of genetics and ethnicity can be largely overlapping.

In addition to intrinsic variation, multiple external factors alter the skin microbiota composition (Table 1; Moskovicz *et al*., 2020). These effects have been attributed to, for example, contact with other people and dogs (Song *et al*., 2013), exposure to environmental microbes (Nielsen and Jiang, 2019; Vandegrift *et al*., 2019), and environmental conditions such as UV radiation (Burns *et al*., 2019). Also, lifestyle‐related variables, such as diet (Brandwein *et al*., 2019), medications (such as antibiotics; Park *et al*., 2020), cosmetics (Bouslimani *et al*., 2019) and contact with housing materials or chemicals (McCall *et al*., 2020), are known to influence the skin microbiome. The effects of external factors can be either temporal or long‐lasting. For example, the residence time of introduced microbes can be affected by the quantity of biomass of the source and the duration of the interactions with it (Vandegrift *et al*., 2019). Healthy skin communities display some resilience to such disturbances and can maintain a stable composition over long periods (Oh *et al*., 2016; Moskovicz *et al*., 2020; Hillebrand *et al*., 2021). However, high frequency or constant interaction with specific external factors can likely lead to more permanent changes in the skin microbiome. As many environmental factors which can affect the skin microbiome have uneven geographical distributions, the place of residence of an individual is also reflected in their skin microbiome composition (Callewaert *et al*., 2020). For example, compositional differences have been observed between urban and rural individuals (Ying *et al*., 2015; Lehtimäki *et al*., 2017), and even between residents of different cities (Sun *et al*., 2019). The compositions of the site‐specific skin microbiomes on a person are dependent on both internal and external factors and their varying effects over that person's lifetime. Therefore, skin microbiomes are highly unique and may even be used to identify an individual (Fierer *et al*., 2010; Schmedes *et al*., 2017). For example, only 2% of species‐level operational taxonomic units (OTUs) were found to be shared among individuals on superficial forearm skin in a study published over a decade ago (Gao *et al*., 2007). Since this seminal study, several promising forensic applications have been identified for skin microbiomes, such as identifying people, their gender or geographical origin, or which individual or body site likely interacted with an object (Neckovic *et al*., 2020). However, a number of challenges remain before skin microbiomes can be routinely used in forensics. For example, sampling and sample handling need to be standardized, reference databases have to be improved, and issues with contamination and legislation need to be resolved (García *et al*., 2020; Neckovic *et al*., 2020).

## Skin microbiomes in health and disease

Humans have coevolved with their skin microbiota (Ross *et al*., 2018). Thus, the communities found on healthy skin generally support the function of the skin as a barrier and prevent the growth of harmful microbes on it (Byrd *et al*., 2018). In addition, skin bacteria seem to play a major role in training our adaptive immune system (Naik *et al*., 2012). This process occurs during childhood and depends on exposure to diverse environmental microbes in the person's living environment (Lehtimäki *et al*., 2017). The combined effects of the whole community on skin function appear to be more than a sum of its parts. For example, the regulatory effects of the whole microbial community were more extensive and pronounced than those of individual microbes in a 3D human skin model (Loomis *et al*., 2021).

As the biodiversity of our living environment is reflected in the biodiversity on our skin, changing exposures of the skin microbiome have predisposed people in industrialized environments to both cutaneous and inflammatory non‐communicable diseases (Prescott *et al*., 2017). Globally, the most common skin diseases associated with microbes, in order of disease‐adjusted life years, are atopic or seborrheic dermatitis, acne vulgaris, urticaria (associated with gut microbiome), psoriasis, viral diseases, fungal diseases, scabies, pyoderma, cellulitis and decubitus ulcer (Table 2; Karimkhani *et al*., 2017; Nabizadeh *et al*., 2017; Ellis *et al*., 2019; Pang *et al*., 2019). Notably, many of these diseases, such as psoriasis and atopic dermatitis, are not directly caused by a single pathogen but are in addition connected to microbe‐immune system imbalances, dysbiosis (Kong *et al*., 2012; Catinean *et al*., 2019), or result from complex microbial interactions affecting the prevalence of virulence genes (Barnard *et al*., 2016). Even diseases caused by single pathogens, such as fungal and viral infections, might involve interactions with other microbes on the skin and the host immune system. For example, commensal *Malassezia* species usually display pathogenic features and cause infections (Grice and Dawson, 2017). The pathogenicity of *Staphylococcus epidermidis*, which is ubiquitous on healthy human skin, is dependent both on the specific strain and the biological context (Brown and Horswill, 2020). Also, skin microbiome dysbiosis has been associated with infections caused by eukaryotic parasites, such as human itch mite (*Sarcoptes scabiei* var. hominis; Bhat *et al*., 2017) and *Leishmania* species (Gimblet *et al*., 2017).

**Table 2:** Associations between common skin diseases and skin microbiota.

Skin disease	Typical body sites	Positive associations (relative abundance ↑)	Negative associations (relative abundance ↓)	References
Atopic dermatitis	Bends of elbows and knees	*Staphylococcus aureus*, (*Staphylococcus epidermidis* in less severe AD)	*Malassezia*, bacterial diversity, eukaryotic diversity	Kong *et al*. (2012); Chng *et al*. (2016); Sun *et al*. (2019)
Acne vulgaris	Sebum‐rich areas (e.g. scalp, face, shoulders)	*Cutibacterium acnes*, *Cutibacterium granulosum*	*Staphylococcus epidermidis*	Barnard *et al*. (2016); Skabytska and Biedermann (2016); Park *et al*. (2020)
Psoriasis	Scalp, elbows, knees, lower back	*Corynebacterium*, *Propionibacterium*, *Staphylococcus*, *Streptococcus*, *Thermomonas*, pathogens (e.g. *Vibrio*)	*Cupriavidus*, *Flavisolibacter*, *Lactobacillus*, *Methylobacterium*, *Schlegelella*, bacteriophages, bacterial diversity	Alekseyenko *et al*. (2013); Wang *et al*. (2020)
Scabies	Multiple sites	*Staphylococcus aureus*, *Streptococcus pyogenes*	Not reported	Whitehall *et al*. (2013); Bhat *et al*. (2017)
Cellulitis	Disrupted sites (e.g. bites, cuts, wounds, dry skin)	*Enterobacter*, *Enterococcus*, *Escherichia coli*, *Pseudomonas aeruginosa*, *Rhodanobacter terrae*, *Staphylococcus aureus*, *Streptococcus pyogenes*	Not reported	Doern *et al*. (1999); Johnson *et al*. (2016)
Decubitus ulcer	Skin over bony areas (e.g. sacrum, heels, elbows, hips)	*Christensenella*, *Enterococcus*, *Eubacterium dolichum*, *Lactobacillus zeae*, *Pseudomonas aeruginosa*, *Staphylococcus aureus*, *Staphylococcus epidermidis*	*Actinobaculum*, *Mycobacterium vaccae*	Wolcott *et al*. (2016); de Wert *et al*. (2020)
*Leishmania* infection	Exposed parts of the body	*Staphylococcus*, *Streptococcus*	Bacterial diversity	Gimblet *et al*. (2017)

The interactions related to skin microbiomes, including environmental exposures and the host immune system, strongly influence the host's overall health (Prescott *et al*., 2017). Unfortunately, mechanistic understanding of these systems has remained incomplete. Advances in sampling, analytical and algorithmic methods currently enable the examination of skin microbiomes at an unprecedented depth (Carrieri *et al*., 2021; Loomis *et al*., 2021). The availability of high‐throughput sequencing (HTS), together with efficient algorithms and computational resources to analyse the massive data sets, has revolutionized our view of the skin microbiome (Liu *et al*., 2021). However, there are several essential considerations in the current methods, which can improve the reliability and reproducibility of the data and analytical results. Furthermore, emerging technologies, such as long‐read sequencing (Amarasinghe *et al*., 2020) and single‐cell genomics (Sharma and Thaiss, 2020), will improve our understanding of these systems. Their application could further develop novel medical diagnostic tools and treatments, lessening the global burden of skin diseases.

## Current analytical approaches

### Study design

**Fig. 2:**
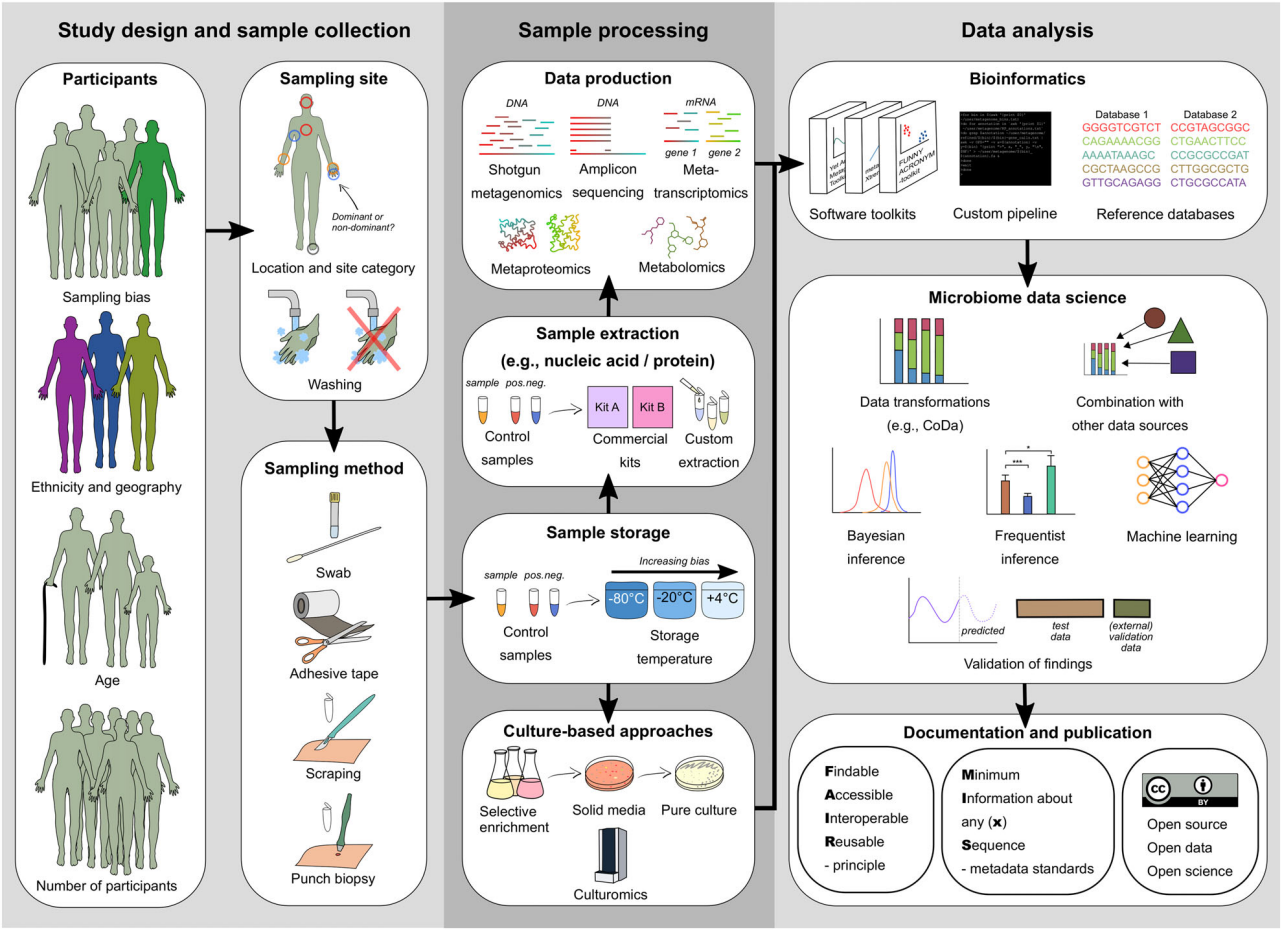
Main steps in a skin microbiome study. Important considerations which can affect the reliability and replicability of the study are outlined at each step. CoDa = Compositional data.

In skin microbiome research, data collection often starts with recruitment of suitable participants and choosing the skin site for sampling (Fig. 2). The exact criteria for choosing participants and their measured medical or demographic variables are highly context‐dependent. However, factors affecting the skin microbiome, in general, should be considered in the study design. For example, the ethnic and geographic origin of the participants, and their age distribution, can influence the results and their generalization from individuals to populations and between different populations (Gupta *et al*., 2017). Statistical power, i.e. the number of participants needed to detect significant differences between the groups of interest, should preferably also be estimated prior to gathering data (La Rosa *et al*., 2012; Kong *et al*., 2017). The exact number of required samples for sufficient power depends on the probability model parameters, the effect size, presence of overdispersion in the data and even the number of reads per sample in sequencing studies (La Rosa *et al*., 2012). While recommendations for the exact number of participants and samples cannot be given, landmark studies in skin microbiomes have usually analysed dozens (e.g. Kong *et al*., 2012) to several hundreds of samples (e.g. Song *et al*., 2013). A number of power estimation tools have also been developed, such as micropower for differences in β‐diversity (Kelly *et al*., 2015), and HMP for differences in relative abundances (La Rosa *et al*., 2012). Insufficiently small sample size is also a current problem in human microbiome studies utilizing machine learning, as it can lead to a biased estimate of a model's predictive performance (Quinn, 2021). After deciding on the study design, necessary measurements based on its specific demands are then collected from the participants. These will further depend on the project goals and limitations of the available equipment or other resources (Jarett *et al*., 2021).

### Sample collection

In the cutaneous sampling, the site and technique choice greatly affect which organisms are enumerated in the sample (Fig. 2; Prast‐Nielsen *et al*., 2019; Bay *et al*., 2020). Because of the high number of external factors that can affect the skin microbiome's composition, exposures of the sampling site to such factors should be controlled before sample collection. For example, participants have been instructed to avoid washing and the use of skin emollients before sampling, often for 12–24 h, and those using systemic or topical antimicrobial compounds have usually been excluded (Kong *et al*., 2017). Both handwashing with soap (Two *et al*., 2016) and swimming in the ocean (Nielsen and Jiang, 2019) can alter the skin microbiome at least for 24 h. Thus, participants should be instructed to avoid washing, bathing and swimming for at least 24 h. While effects of washing might persist over longer times, longer time limits would likely be impractical for recruitment of participants. However, the use of antibacterial soap affects the skin microbiome at least for 2 weeks (Yu *et al*., 2018). The use of any antibacterial compounds in soaps and emollients should therefore be more carefully controlled, and participants using such products should also be excluded. Avoiding skin washing also has the added benefit of somewhat increasing the naturally low biomass of the skin microbiome (Bjerre *et al*., 2019). Samples with low microbial biomass are, for example, highly prone to DNA contamination from external sources. Methods to control and account for such contamination have been suggested, such as randomizing sample types and treatment groups during sample collection (Eisenhofer *et al*., 2019).

In most studies, the body site for sampling has been defined as a distinct anatomical location, such as the volar forearm or forehead (Fig. 1). However, care should be taken when defining the exact sampling site, as, e.g. handedness (which hand is dominant) can lead to differences in microbiota composition at an anatomically identical sampling site (Lehtimäki *et al*., 2017). The conditions within a specified body site might also vary with corresponding small‐scale differences in the microbiome (Bjerre *et al*., 2019). This type of high‐grain spatial organization of the microbiome might explain some of the sampling bias observed with the most common sampling methods (Prast‐Nielsen *et al*., 2019). Micrometre‐scale spatial organization of bacteria on tongue dorsum has been recently described using multiplexed fluorescence spectral imaging (Wilbert *et al*., 2020). Similar approaches applied to skin microbiomes could likely elucidate these poorly known high‐grain spatial patterns. For example, a recent study on spatial organization of *C*. *acnes* on facial skin revealed that individual pores are dominated by single lineages, enabling the stable coexistence of diverse strains of the bacterium (Conwill *et al*., 2022).

Currently, the most common sampling methods for skin microbiomes are swabbing, tape‐stripping, scraping and punch biopsy (Fig. 2; Bjerre *et al*., 2019; Prast‐Nielsen *et al*., 2019). These techniques differ, for example, in their invasiveness and discomfort level, depth of skin sampled, amount of biomass recovered and sampling bias (Table 3). Recent comparisons between these methods for HTS studies exist, e.g. for swabbing, tape stripping and scraping (Chng *et al*., 2016), swabbing and tape stripping (Ogai *et al*., 2018), swabbing and scraping (Bjerre *et al*., 2019), and swabbing and biopsies (Prast‐Nielsen *et al*., 2019). Substantial differences can also exist within these methods. For example, the material of the swab (Warnke *et al*., 2014) and adhesive tape (Ogai *et al*., 2021) have measurable effects on the recovery of skin microbes. Overall, most sampling methods produce similar results for the abundant taxa, but differences can be significant for rare low abundance taxa. Thus, both the sampling area and the technique to be used are essential questions and should be weighted accordingly. For example, either tape stripping or punch biopsy should be used in studies where fine‐scale spatial patterns on the skin need to be examined, as they technically enable the preservation of spatial patterns of the skin microbiome (Table 3). Several sampling methods can be used simultaneously, but this will lead to higher sampling effort and costs. More intensive sampling can also increase the participants' discomfort. However, this can be justified, e.g. for the use of both swabbing and biopsies when studying diseases in the dermis (Prast‐Nielsen *et al*., 2019). Importantly, consistent sampling of a well‐defined site with similar sampling methods needs to be applied to ensure the replicability and comparability of the results. Here, standardization of the size and position of the swabbed area or tape strip could likely be used to lower sampling bias, at least within a single study. Factors which could help with standardization are, for example, uniform sampling equipment (same size and manufacturer), reporting of the exact size of the sampling area (such as 5 × 5 cm) and location (e.g. volar forearm on non‐dominant side), and accounting for interpersonal differences (e.g. applied pressure, time and swiping pattern). While some of these factors can be assessed in data analysis, further research is still needed to develop sampling tools and methods with reduced bias.

**Table 3:** Comparison of the four most common methods of sample collection for skin microbiome analysis.

Method	Description	Depth sampled	Invasive	Requires expert user	Amount of biomass	Pros	Cons
Swabbing	Skin is swabbed with a sterile (wetted) fibre‐tipped swab	Epidermis	No	No	+	Widely used method increases comparability	Only samples the top layer of skin
Tape stripping	Sterile adhesive tape is attached to the skin and removed	Epidermis	No	No	+	Preserves spatial structure in two dimensions	Can be unsuitable for fragile skin
Scraping	A cup or scalpel is used to scrape the skin to obtain a sample	Epidermis	No	No	++	Samples slightly deeper than swabbing and tape stripping	Can be unsuitable for fragile skin. High number of human cells in samples
Punch biopsy	A small piece of skin tissue is removed with a sharp, circular and hollow instrument	Epidermis and dermis	Yes	Yes	+++	Preserves spatial structure in three dimensions	Not well‐suited for exposed skin surfaces. Very high number of human cells in samples

### Sample processing

After the sample collection, several routes can be taken to examine the microbial community in the samples. Notably, all processing immediately after sampling should consider sample exposure time to different ambient temperatures to minimize variation between the samples (Fig. 2). Storage temperatures have a limited effect on the microbial diversity for molecular analyses (Lauber *et al*., 2010). However, freeze–thaw cycling should be avoided, as it affects the community profiles (Cuthbertson *et al*., 2015). Although storage of microbiota samples is often recommended at −80°C, storage of skin swab samples at this low temperature can still affect downstream molecular analyses (Klymiuk *et al*., 2016). Cryogenic storage of tissue samples in liquid nitrogen (−196°C) can accurately preserve DNA and RNA for over a decade (Kelly *et al*., 2019), but we are not aware of similar studies on skin microbiomes, likely due to the brief history of the field. While further research is still needed, increasingly low temperatures are probably better also at preserving all types of skin microbiome samples (biopsies, swabs and strips) as long as the device materials tolerate this.

In addition to storage temperatures, the use of stabilizing agents such as RNA*later*® and DNA/RNA Shield™ can introduce bias to the analyses (Menke *et al*., 2017; Angebault *et al*., 2018; Hallmaier‐Wacker *et al*., 2018). A recent comparison of storage methods applicable to resource‐limited settings found that their effect on the differences in microbiota composition was lower than that of the collection method or site (Manus *et al*., 2022). However, the low biomass of skin swab samples also appears to make them more susceptible to disruption during storage than faecal and saliva samples (Marotz *et al*., 2021). Best practices might also depend on practical considerations, such as cost‐effectiveness and availability of low‐temperature storage (Marotz *et al*., 2021). Thus, sample storage should be carefully evaluated based on current research, and the use of control samples undergoing similar treatment as the skin samples is strongly recommended to minimize possible bias.

Currently, most skin microbiome studies utilize solely culture‐independent methods based on high‐throughput sequencing instead of isolating and culturing single organisms from the samples (Fig. 2). For this purpose, nucleic acids (DNA or RNA), proteins, or metabolites are extracted from the samples and analysed. The choice of the extraction method can significantly affect the downstream analyses. For example, notable differences exist between commercial DNA extraction kits (Bjerre *et al*., 2019). In addition, contamination within the extraction kits, laboratory reagents, equipment and also cross‐contamination from other samples and sequencing runs can be an issue in molecular analyses of low biomass samples, such as the skin microbiome (Salter *et al*., 2014; Kim *et al*., 2017; Eisenhofer *et al*., 2019). Therefore, proper negative and positive controls with defined microbial composition should be included throughout sample processing to reveal possible biases and account for their effects (Hornung *et al*., 2019). Specific guidelines for conducting and reporting low biomass microbiome studies have also been recently developed (Eisenhofer *et al*., 2019). These guidelines include, e.g. the use of clean environments and protective clothing, and decontaminating consumables, containers and reagents (for further discussion, see Eisenhofer *et al*., 2019). In skin microbiomes, the amount of host DNA can be upwards of 90% of total extracted DNA, which can be challenging for downstream analyses, as a large part of the sequencing effort in shotgun metagenomic sequencing is spent on the human genome instead of the microbiome (Bjerre *et al*., 2019). A customized chemical extraction method with propidium monoazide decreased human DNA reads by an order of magnitude and outperformed several commercial kits with a low taxonomic bias when analysing saliva samples (Marotz *et al*., 2018). A recent study used a commercial kit for host DNA depletion to reduce human reads from 92% to 32% in skin samples, but the taxonomic bias and problems with library construction were deemed too high for practical applications (Ahannach *et al*., 2021a). In the same study, propidium monoazide and the two commercial kits appeared to also decrease the relative abundances of Gram‐negative microbes in 16S rRNA gene amplicon sequencing of skin samples. Thus, host DNA depletion cannot be currently recommended for skin microbiome research because the observed taxonomic bias cannot be justified purely by decreased sequencing costs.

### Culture‐based approaches

Culturing selects microbes best adapted to the utilized growth conditions, such as nutrient availability, pH and temperature (Bonnet *et al*., 2020). This focuses culture‐based analyses on the cultivable minority of skin microbes. For example, culturing detected only 16% of the OTUs found with 16S rRNA sequencing in chronic wound infections, while sequencing detected 85% of the cultured strains (Rhoads *et al*., 2012). Overall, culture‐independent methods enable a more detailed characterization of the skin microbiome than culture‐based methods (Table 4; Timm *et al*., 2020) and should thus be used for diversity analyses. However, culturing provides an unparalleled opportunity to study an individual microbial strain's growth, metabolism and biological interactions in controlled conditions (Thrash, 2019). Standardized culture‐dependent methods are also often used in clinical microbiology (Boers *et al*., 2019). For example, the susceptibility of microbial strains to antibiotics is difficult to estimate with culture‐independent methods (Vasala *et al*., 2020). Culture‐based studies also widely benefit culture‐independent studies. For example, culturing enables the identification of new species for improving reference databases (Nasko *et al*., 2018). Cultured species and strains can also be deposited in culture collections, enabling the in‐depth study of microbes detected with culture‐independent methods (Timm *et al*., 2020). Furthermore, culturomics has emerged as a promising high‐throughput method to identify and characterize novel microbial species while simultaneously obtaining them in culture (Lagier *et al*., 2018). This approach consists of inoculating samples into varying conditions to obtain even tens of thousands of individual bacterial colonies, rapidly and cheaply identifying known species with matrix‐assisted laser desorption ionization time‐of‐flight mass spectrometry and characterizing the unidentified species with 16S rRNA amplicon sequencing. This method has also been successfully applied on human skin for bacteria (Cassir *et al*., 2015) and fungi (Leong *et al*., 2021).

**Table 4:** Comparison of several culture‐based and culture‐independent approaches for analysing skin microbiomes.

Category	Method	Costs per sample			Coverage for taxonomic characterization	Resolution for taxonomic characterization			Functional analysis		Relevant applications			Other pros	Other cons	References	
		Labour	Assay	Analysis		Genus	Species	Strain	Prediction	Measurement	Characterization of novel microbes	Identification of known microbes	Other applications			
Culture‐based	Isolation of pure cultures	+++	+	+	+	X	X	X	NA	+++	X	X	Mechanistic studies	Well‐established standard methods	Organisms can grow very slowly or require complex or unknown growth media	Thrash (2019); Timm *et al*. (2020)
													3D skin models	Extremely high reliability		
	Culturomics	+	+	+	Clinically relevant taxa (+)	X	X	X	NA	+	X	X		High potential for further method development	Databases are still very limited	Lagier *et al*. (2018); Rahi and Vaishampayan (2020)
														Enables collection of characterized strains		
Culture‐independent	16S rRNA /ITS amplicon sequencing	+	+	++	Bacteria and archaea (++)	X	X	−	+	−	−	X		Well‐established standard methods	Method development might have plateaued	Klindworth *et al*. (2013); Pollock *et al*. (2018); Johnson *et al*. (2019); Milani *et al*. (2020)
															Many sources of bias	
	Full‐length 16S rRNA amplicon sequencing	+	++	++	Bacteria and archaea (++)	X	X	X	+	−	−	X		Improved resolution over short‐read 16S rRNA amplicon sequencing	Availability of long‐read sequencing might be low	Johnson *et al*. (2019)
															Many sources of bias	
	Deep shotgun metagenomic sequencing	+	+++	+++	+++	X	X	X	+++	−	X	X		Most comprehensive characterization of the whole microbiome	High fraction of sequencing effort in skin microbiome is spent on host genome	Quince *et al*. (2017); Tett *et al*. (2017); Liu *et al*. (2021)
	Shallow shotgun metagenomic sequencing	+	++	++	+++	X	X	−	++	−	−	X		Higher resolution compared to 16S rRNA amplicon sequencing with similar cost	High fraction of sequencing effort is spent on host genome	Hillmann *et al*. (2018, 2020); Cattonaro *et al*. (2020)
															Might be unsuitable for skin microbiomes	
	Metatranscriptomics	++	+++	+++	+++	X	X	−	NA	Gene expression (+)	−	Metabolically active (X)	Studying changes in community gene expression	Simultaneous analysis of host and microbiome transcripts	Simultaneous analysis of host and microbiome transcripts (if not wanted)	Bashiardes *et al*. (2016); Shakya *et al*. (2019)
														Complements other analyses		
	Metaproteomics	++	+++	+++	Active microbiota (+++)	X	−	−	NA	Active metabolism (+)	−	−	Study of active metabolism	Complements other analyses	Coverage of databases still needs improvement	Mills *et al*. (2019); Li and Figeys (2020)

	Metabolomics	++	+++	+++	Only in combination with other *omics	−	−	−	NA	Microbial and host metabolites (+)	−	−	Study of active metabolism	Complements other analyses Enables tracking of stable isotopes	Data analysis is highly complex	Ribbenstedt *et al*. (2018); Emwas *et al*. (2019)	

### High‐throughput sequencing

HTS has revolutionized our understanding of the microbial world (Blaser, 2014; Kanangat and Skaljic, 2021; Ahannach *et al*., 2021b), as it has enabled the detection and characterization of the difficult‐to‐culture majority of microbes in a wide range of environments, including the human body (Boers *et al*., 2019). Culture‐independent methods such as amplicon sequencing and shotgun metagenomics are currently utilized in most skin microbiome studies (Fig. 2; Kong *et al*., 2017). Amplicon sequencing is most commonly used to profile the archaeal and bacterial communities. Here, a section covering a few hundred nucleotides of the highly conserved 16S ribosomal RNA gene is amplified and sequenced with HTS (Klindworth *et al*., 2013). Recently, amplicon sequencing of the nuclear ribosomal internal transcribed spacer (ITS) region has also been used to obtain (sub)species resolution of bacterial communities (Milani *et al*., 2020). ITS‐based profiling might thus serve as a viable alternative for 16S rRNA‐based profiling of bacterial communities in the future. For fungi, various targets in the ITS or small and large subunit ribosomal RNA genes (SSU/LSU) are used (Nilsson *et al*., 2019). As a cost‐effective and relatively simple method, amplicon sequencing (i.e. metabarcoding) has been widely used for characterizing the microbiomes' taxonomy and diversities. The functional potential of the community can also be predicted with amplicon sequencing based on the taxonomic identification of the organisms, but the accuracy of this approach is limited (Liu *et al*., 2021). The taxonomic resolution and other biases in these analyses are also affected by choice of primers, targeted variable regions, sequencing technology and downstream data analysis, and it is likely that no ‘best practices’ exist for 16S rRNA sequencing (Pollock *et al*., 2018). For example, the use of amplicon sequence variants has been recently studied as a more accurate alternative to OTUs in microbiota profiling (Callahan *et al*., 2017). However, all amplicon‐based approaches are prone to similar biases (Nearing *et al*., 2021). Using either approach can result in the same biological conclusions with 16S rRNA amplicon sequencing (Moossavi *et al*., 2020), and the same issues apply to fungal amplicon sequencing (Nilsson *et al*., 2019). For example, specific primer sets for the 16S rRNA gene can sufficiently characterize skin bacteria (Castelino *et al*., 2017), but most universal primer pairs greatly overlook archaeal diversity (Bahram *et al*., 2019).

Furthermore, no fractional part of the 16S rRNA gene enables species‐level resolution for bacterial identification (Johnson *et al*., 2019). Amplicon sequencing with short‐read HTS platforms is thus not useful beyond genus‐level resolution for archaea and bacteria. Thus, the choice of primers and the sequenced region of the target gene is always a compromise, which should be based on coverage of likely important taxa, and desired comparisons to previous studies and data.

Shotgun metagenomic sequencing means non‐targeted sequencing of the total DNA in a sample (Quince *et al*., 2017). This is a powerful method that can potentially characterize all community members and their functional genes. Compared to amplicon sequencing, shotgun metagenomics can provide strain‐level taxonomy of the organisms (Tett *et al*., 2017) and covers all components of the microbiome, including the virome and mobilome (mobile genetic elements; Carr *et al*., 2021). However, these benefits are offset by the increased sequencing effort and cost, the required biomass in the sample and more complex data analysis (Table 4; Liu *et al*., 2021). The use of shotgun metagenomics in skin microbiomes is further complicated by the high amount of host DNA in the samples. The host DNA sequences can be removed during data processing, but a large part of the sequencing effort is spent on sequencing of the human genome, which is usually an undesired outcome. However, both the cost of sequencing and template requirements have constantly been decreasing. In addition, the broader application of host DNA depletion methods could further increase the applicability of shotgun sequencing. The amount of bioinformatic methods available for shotgun metagenomic data is large enough to seem overwhelming for researchers entering this field. However, these can be roughly divided into assembly (or contig) based and read based approaches (Liu *et al*., 2021).

Briefly, in assembly‐based methods, the sequencing reads are assembled into long contiguous pieces of DNA sequence, which are then matched against pre‐existing databases to identify genes and organisms. The contigs can also be clustered to genomic bins based on, e.g. tetranucleotide frequency and uneven abundance of the contigs in different samples. The binning produces draft metagenome‐assembled genomes (MAGs) and has proven to be a highly potent tool to examine the diversity of the skin microbiome (Li *et al*., 2021). Such analyses, however, require expensive deep sequencing of the samples and computational effort for assembly and binning, making them unsuited for researchers with limited budgets and resources, especially if a high number of samples are involved.

Read‐based methods characterize the taxonomic and functional diversity of the community by aligning individual reads from HTS to reference databases. The matching is usually based on clade‐specific marker genes, and in some methods, the taxonomic coverage has included all prokaryotic and eukaryotic microbes and viruses (Truong *et al*., 2015). While these tools generally have higher taxonomic coverage and lower bias than amplicon sequencing, their results are, however, dependent on the quality of the available marker gene databases (Lugli *et al*., 2019). Recently, shallow shotgun sequencing has shown to be a cost‐effective alternative to 16S rRNA amplicon sequencing (Hillmann *et al*., 2018). It enables taxonomic profiling (Hillmann *et al*., 2020) but not contig or genome assembly (Cattonaro *et al*., 2020). Therefore, for genome assembly, which often requires immense sequencing effort, associated costs can be lowered by combining short‐read sequencing with emerging long‐read sequencing technologies (Sanders *et al*., 2019).

### Other culture‐independent approaches

In addition to amplicon and shotgun sequencing, several other omics methods have been used to profile skin microbiomes without laborious and time‐consuming culturing steps. The three approaches introduced here, metatranscriptomics, metaproteomics and metabolomics, focus on analysing the function of the microbial community at the time of sampling (Fig. 2; Table 4; Aguiar‐Pulido *et al*., 2016). This information is invaluable in understanding how the microbial ecosystem reacts to different stimuli and interactions within it and between the host. Similar to patterns detected with metagenomics, the other omics tools can also produce diagnostically useful signals, such as skin microbiota‐associated plasma metabolites as biomarkers for psoriasis (Chen *et al*., 2021).

In metatranscriptomics, mRNA present in the samples is extracted and reverse‐transcribed to complementary DNA to facilitate its analysis and long‐term storage (Shakya *et al*., 2019). In contrast to above‐mentioned DNA‐based analyses, metatranscriptomics directly profiles actively transcribed genes within the community, which are a proxy for its active metabolism at the moment of sampling. The current limitations of this method are the low coverage of existing reference databases, increased effort in sample preparation, high risk of contamination and mRNA degradation, and high cost (Sandhu *et al*., 2019). Despite these issues, metatranscriptomics has a high potential for elucidating the response of the skin microbiome to various conditions related to skin diseases. For instance, it has been used to show that specific pathways in *Cutibacterium acnes* (formerly *Propionibacterium acnes*) respond to vitamin B12 supplementation and are involved with the development of acne (Kang *et al*., 2015).

In metaproteomics, proteins extracted from a sample are most often separated with liquid chromatography and detected with mass spectrometry (Wilmes and Bond, 2006). This analysis produces spectral data which are referenced to pre‐existing databases to identify peptides and proteins, their abundances, and the presence of any post‐translational modifications, such as phosphorylation and acetylation. Metaproteomics is complementary to the other omics methods as it provides very accurate information on the function of the community at the time of sampling. However, it similarly suffers from laborious preprocessing steps, lacking reference databases, and challenges in data processing because of the high amount of data produced (Li and Figeys, 2020). It is also not as well suited for skin microbes since it requires a somewhat high amount of biomass, often obtained through enrichment steps (Petriz and Franco, 2017). However, recent developments have led to a more simplified laboratory and data production workflow, which might be more applicable for skin studies (Heyer *et al*., 2019).

In metabolomics, the cellular metabolic products in the skin or other tissue samples are extracted, characterized and quantified (Ribbenstedt *et al*., 2018). Most commonly, an extract of the metabolites is separated into components with liquid or gas chromatography (L/GC), and the compounds are characterized with mass or nuclear magnetic resonance spectrometry (MS/NMR; Ribbenstedt *et al*., 2018; Emwas *et al*., 2019). In non‐targeted metabolomics, the whole range of non‐identified compounds in the sample is correlated with an experimental condition. In the targeted approach, only specific metabolites matching a standard sample are detected. This approach is highly adept at detecting small compounds produced by the microbiota and is often combined with amplicon or shotgun metagenomic sequencing. Similarly to metaproteomics, the high cost, laborious sample preparation and sparse reference databases have however limited its applications in microbiomes (Sandhu *et al*., 2019). In skin microbial communities, metabolomics has been used to characterize biomarkers of healing wounds and their connections with specific bacteria in the associated microbial community (Ashrafi *et al*., 2020). Metabolomic analysis of blood plasma integrated with metagenomic profiling of the skin microbiome has also detected microbiota‐associated metabolites with potential as diagnostic markers for psoriasis (Chen *et al*., 2021).

### Data analysis

A large variety of computational techniques and tools have been developed to process amplicon and shotgun sequencing and other types of omics data from microbiomes. Several choices need to be made in the bioinformatic and data science steps of a skin microbiome project (Fig. 2), and the number of available options is immense. Invaluable microbiome and metagenome analysis software and toolkits are available today, such as Anvi'o (Eren *et al*., 2021), bioBakery 3 (Beghini *et al*., 2021), QIIME2 (Bolyen *et al*., 2019), phyloseq (McMurdie and Holmes, 2013) and the R/Bioconductor ecosystem (Gentleman *et al*., 2004). They provide standardized solutions for most statistical and data analytical challenges related to the relevant omics data types while allowing considerable flexibility to design and implement custom workflows (Shetty and Lahti, 2019). Following the general developments in human microbiome studies, skin microbiome research increasingly employs multiple parallel omics assays, complementing metagenomic measurements with transcriptomics, metabolomics, single‐cell sequencing and other omics technologies. Thus, appropriate statistical techniques (Sankaran and Holmes, 2019b) and ways to organize complex multi‐assay data sets to facilitate smooth quantitative analyses are needed. Data science solutions, such as the *TreeSummarizedExperiment* data container (Huang *et al*., 2021), have been recently proposed as a dedicated approach to deal with such data aggregation tasks. While many tools can produce robust results, each method is prone to specific biases (Lugli *et al*., 2019; Moreno‐Indias *et al*., 2021). Therefore, the evaluation of skin microbiome studies should begin with the description of participants and related metadata. This should continue to detailing of the data production, processing and analysis, through interactive and reproducible workflows and electronic notebook environments to ensure complete transparency, reproducibility and reusability of the data analytical work (Ragan‐Kelley *et al*., 2013; Callahan *et al*., 2016; Rule *et al*., 2019).

While a thorough assessment of the methodology for microbiome data analysis is beyond the scope of this study, we can recommend reviews on the available tools and give recommendations for different purposes (Knight *et al*., 2018; Gardner *et al*., 2019; Shetty and Lahti, 2019; Liu *et al*., 2021). Challenges in the study of skin microbiomes, such as the large quantity and dimensionality of the data, and its compositionality (Gloor *et al*., 2017), are similar to those encountered in the study of other human microbiomes. Notably, compared to other body sites, such as sites along the gastrointestinal tract, the high variability of skin microbiome composition and the low biomass on skin adds further uncertainty in the analyses. Thus, probabilistic and latent variable techniques could further aid in quantifying associated uncertainties and improving the robustness of analyses when sample sizes or read counts are small, and uncertainty is high (Äijö *et al*., 2018; Metwally *et al*., 2018; Sankaran and Holmes, 2019a; McGhee *et al*., 2020; Marcos‐Zambrano *et al*., 2021). Furthermore, the reproducibility crisis in science (Baker, 2016) also reaches the study of human microbiomes, especially with the recent surge in studies utilizing machine learning (Moreno‐Indias *et al*., 2021). The widespread use of improper practices can be seen, for example, in gut microbiome studies utilizing machine learning (Quinn, 2021). These issues can likely also affect skin microbiome studies. Thus, more robust practices should be adopted in machine learning, such as using nested cross‐validation and completely separating the training and test (or validation) data (Vabalas *et al*., 2019).

To facilitate progress in skin microbiome research through adoption of open science practices, we recommend that the data and analysis code be made findable, accessible, interoperable and reusable (FAIR; Moreno‐Indias *et al*., 2021). For these purposes, minimum information about any sequence metadata standards has recently been developed (Bowers *et al*., 2017), and adopted by several databases, such as NCBI and EMBL. The use of MixS and other suitable metadata standards is recommended because these standards cover the most critical parts of the data production and processing in microbiome studies and further the FAIR principles (Vangay *et al*., 2021).

## Perspectives

The understanding of the structure and function of the skin microbiome has greatly increased during the last decade. This progress has been primarily fuelled by modern omics tools, especially amplicon sequencing and metagenomics. Here, we have identified several technological advancements and recent maturation of existing tools which could grant even further insights into the skin microbiomes and facilitate their future practical applications.

A number of future applications based on analysis of skin microbiomes can be envisioned in the field of forensics (Ahannach *et al*., 2021b). Such applications are possible, because humans leave traces of their skin microbial signature on touched items (Knights *et al*., 2011). Skin microbial communities are also stable over long periods on the same site on an individual (Oh *et al*., 2016). Thus, skin microbiomes might be proven useful in tracking contacts between people and objects to provide trace evidence for criminal cases and personal identification (Tozzo *et al*., 2020). Microbial source tracking has been developed and utilized primarily for tracking faecal contamination and pathogens in water (Boehm *et al*., 2013). Forensic application of skin microbiomes has recently been reported, utilizing both metagenomics sequencing (McGhee *et al*., 2020) and 16S rRNA amplicon sequencing data (Carter *et al*., 2020). High‐resolution features of the skin microbiome appear to be most helpful in personal identification. Analysis of rare prokaryotic taxa on forehead skin with 16S rRNA amplicon sequencing correctly identified 78% of 89 participants when several samples per person were analysed (Watanabe *et al*., 2018). Nucleotide diversity of subsets of clade‐specific markers from shotgun metagenomic sequencing was analysed to achieve, e.g. 96% identification accuracy on palm skin samples from 12 participants (Schmedes *et al*., 2017). Recently, amplicon sequencing of prokaryotic clustered regularly interspaced short palindromic repeats provided 95% accuracy in identifying 14 participants (Toyomane *et al*., 2021). Despite these promising results, there remain some practical and legal considerations that need to be addressed before the widespread adoption of microbiome‐based methods in forensics (Clarke *et al*., 2017; García *et al*., 2020). For example, to the best of our knowledge, population‐scale studies on the forensic utility of skin microbiome analysis have yet to be reported. Furthermore, addressing issues related to bias introduced in, e.g. sampling, sample handling and storage and standardization of analyses, highlighted in our review, would also likely increase the applicability of skin microbiomes in the forensic field.

While most of the current data in skin microbiomes are produced with potent HTS methods, such as Illumina's MiSeq and Thermo Fisher's Ion Torrent, these technologies have produced read lengths only of a few hundred base pairs. This can be problematic for amplicon and shotgun metagenomic sequencing because short amplicons on the 16S rRNA gene allow only genus‐level taxonomic identification, and genome assemblies based on short reads can be highly fragmented. Long‐read technologies, such as Sequel II from Pacific Biosciences, and MinION from Oxford Nanopore Technologies feature up to 50 kbp (PacBio) or 100 kbp (Nanopore) library insert sizes with average read lengths of over 10 kbp (Amarasinghe *et al*., 2020). Also, the previously high error rates of these methods are currently down to single digits (<1%) for PacBio and (<5%) Nanopore. This has made them highly useful in, e.g. hybrid genome assembly when combined with high‐quality short‐read data (Xie *et al*., 2020). The use of long‐read platforms should be further explored for full‐length 16S rRNA amplicon sequencing for strain‐level characterization of communities (Johnson *et al*., 2019). In long‐read sequencing, it should be noted that fragmentation of extracted DNA can present further issues (Maghini *et al*., 2021). Thus, the potential of high‐molecular‐weight DNA extraction (Maghini *et al*., 2021) and host DNA depletion (Marotz *et al*., 2018) could be investigated in the context of skin microbiomes to improve the template quality, especially for long‐read shotgun metagenomics.

In read‐based metagenomics, the originating species for functional genes is often uncertain or completely unknown. Furthermore, in assembly‐based metagenomics, the contigs and MAGs are only average representations of the genomic content in the actual microbes in the samples. These issues could likely be addressed with single‐cell metagenomics, where the genomic contents of individual microbial cells isolated from the sample are analysed separately (Xu and Zhao, 2018; Sharma and Thaiss, 2020). Some of the first applications of such single‐amplified genomes (SAG) in microbiome research are highly promising. For example, the analysis of SAGs from uncultured strains enabled a detailed reconstruction of dietary fibre fermentation pathways in the mouse gut (Chijiiwa *et al*., 2020). However, like other promising methods, several issues need to be addressed before the widespread adoption of single‐cell metagenomics. For example, in skin microbiomes, the isolation of individual microbial cells from the samples using microfluidics (Tan and Toh, 2020) could aid with the issue of extensive host DNA contamination in metagenomic analyses.

The data analysis in host‐associated microbiomes, such as the human skin microbiome, is currently dominated by traditional statistics, including distance‐based methods (Ruuskanen *et al*., 2021). However, these often do not account for many key features of microbiome profiling such as compositionality, heteroscedasticity, non‐linear relationships, hierarchical and spatial structures, and functional redundancy. In addition, skin microbiomes display extensive spatial variation between individuals because of their close connection with the environment (Vandegrift *et al*., 2019). Spatial information alone could likely also serve as a proxy for variables with uneven spatial distributions, such as differences in surrounding vegetation and lifestyle‐related factors. Thus, spatial modelling methods could likely improve their use in microbiomes in disease diagnosis and prediction.

## Concluding remarks

The increased application of long‐read sequencing and single‐cell metagenomics is now shifting the research focus from mere correlations towards a more mechanistic understanding of the skin microbiomes. Understanding the dynamics of skin microbiomes at the mechanistic level is a prerequisite for future applications in forensics and medicine. However, the limited reproducibility has been slowing down the overall progress in skin microbiome studies. The reproducibility of research could be improved with standardization of the methods, combined with transparent reporting and open research practices (Moreno‐Indias *et al*., 2021). More diverse data sets need to be collected to account for differences in host‐associated and external factors affecting skin microbiomes, such as ethnicity, sex, age and the environment.

### Author Contributions

M.O.R., D.V., R.P., A.R., R.A. and L.L. designed the work. M.O.R., D.V. and R.P. drafted the manuscript. M.O.R. drew the figures. A.R. and E.M. provided critical feedback and suggestions. R.A. and L.L. supervised the work. All authors edited and approved the final version.
